# Alprazolam-related deaths in Scotland, 2004–2020

**DOI:** 10.1177/02698811221104065

**Published:** 2022-08-01

**Authors:** John Martin Corkery, Amira Guirguis, Stefania Chiappini, Giovanni Martinotti, Fabrizio Schifano

**Affiliations:** 1 Psychopharmacology, Drug Misuse and Novel Psychoactive Substances Research Unit, School of Life and Medical Sciences, University of Hertfordshire, Hertfordshire, UK; 2 Swansea University Medical School, Swansea University, Swansea, UK; 3 Department of Neuroscience, Imaging and Clinical Sciences, “G. D’Annunzio” University, Chieti, Italy

**Keywords:** Alprazolam, Xanax, Scotland, United Kingdom, deaths, fatalities, mortality

## Abstract

**Background:**

The benzodiazepine drug alprazolam, a fast-acting tranquiliser, cannot be prescribed on the National Health Service in the United Kingdom. Illicit alprazolam supply and consumption have increased. Concern about increasing numbers of alprazolam-related fatalities started circulating in 2018. However, statistics on this issue are very limited. This study examined patterns in such mortality in Scotland.

**Methods:**

Statistics on deaths where alprazolam was mentioned in the ‘cause of death’ were obtained from official mortality registers. Anonymised Scottish case-level data were obtained. Data were examined in respect of the characteristics of decedents and deaths using descriptive statistics.

**Results:**

Scotland registered 370 deaths in 2004–2020; 366 of these occurred in 2015–2020: most involved males (77.1%); mean age 39.0 (SD 12.6) years. The principal underlying cause of death was accidental poisoning: opiates/opioids (77.9%); sedatives/hypnotics (15.0%). Two deaths involved alprazolam alone. Main drug groups implicated: opiates/opioids (94.8%), ‘other benzodiazepines’ (67.2%), gabapentinoids (42.9%), stimulants (30.1%), antidepressants (15.0%). Two-thirds (64.2%) involved combinations of central nervous system (CNS) depressants.

**Discussion:**

Alprazolam-related deaths are likely due to an increasing illicit supply. The fall in deaths in 2019–2020 is partially due to increased use of designer benzodiazepines. Treatment for alprazolam dependence is growing. Clinicians need to be aware of continuing recreational alprazolam use. When such consumption occurs with CNS depressants, overdose and death risks increase.

**Conclusions:**

More awareness of alprazolam contributing to deaths, especially in conjunction with other CNS depressants, is needed by consumers and clinicians. Improved monitoring of illicit supplies could identify emerging issues of medicines’ abuse.

## Introduction

Alprazolam (8-chloro-1-methyl-6-phenyl-4*H*-[1,2,4]triazolo[4,3-*a*][1,4]benzodiazepine) belongs to the triazolobenzodiazepine class of drugs (Dawson et al., 1984). It acts on the gamma-aminobutyric acid (GABA)_A_ receptor in the brain, producing a depressant effect thereby reducing anxiety (Dawson et al., 1984). It is commonly used to treat anxiety, panic disorders, generalised anxiety disorder, as well as depressive symptoms (van Marwijk et al., 2012).

Pharmaceutical companies market alprazolam under many brand names, including Xanax in the United Kingdom (UK), where only 0.25 and 0.50 mg tablets are legally available (BNF, 2020). In the UK, alprazolam is a Class C drug under the Misuse of Drugs Act 1971 and is in Schedule 4 Part 1 of the Misuse of Drugs Regulations 2001 as a licenced prescription-only medicine. It is not approved for any indication and thus is unavailable on the National Health Service (NHS) (see NHS Electronic Drug Tariff (2018)). However, it can be obtained via a private prescription.

## Effects of alprazolam

Bioavailability of alprazolam is high (80%), peak levels being achieved 1–2 h after oral administration (Smith et al., 1984). It is metabolised in the liver, largely by cytochrome P450 3A4 (Dresser et al., 2000). It has a half-life of 6–12 h; metabolites and remaining alprazolam being excreted in urine (Dawson et al., 1984). Effects start within 20–40 min, with peak levels being achieved in 1–2 h (Sheehan et al., 2007; Smith et al., 1984). Recreational users report experiencing effects for 4–6 h, with residual effects lasting up to 12 h (Erowid, 2017).

Alprazolam can cause a range of side-effects including central nervous system (CNS) depression, especially in conjunction with other substances such as opiates/opioids; withdrawal problems (Ait-Daoud et al., 2018) such as rebound anxiety (Verster and Volkerts, 2004), panic disorder (Bashir and Swartz, 2002) and increased suicidal ideation and attempts (Jonas and Hearron, 1996; Mahendran and Liew, 2010). Alprazolam also gives rise to increased extracellular dopamine concentrations in the striatum and increased serotonin levels (Ait-Daoud et al., 2018). Therefore, it appeals to recreational drug users. Xanax is the most counterfeited form of alprazolam (LGC/Eurofins, 2017–2020).

## Abuse potential

Although typically taken orally, whether as tablets or crushed and swallowed as a ‘bomb’ or ‘parachute’ (Drugs-Forum, 2008) there are also some reports of alprazolam being taken in other ways: injection (Wang and Chew, 2006), snorted (Sheehan et al., 1991) or sublingually on a blotter (Microgram, 2008). Alprazolam is also offered for sale online in powder form and is even used in UK penal establishments (Higgins et al., 2019).

Medically recommended therapeutic doses for short-term use in anxiety are 0.25–0.50 mg three times daily (EMC, 2020). Recreational doses suggested on drug user fora are typically much higher: light (0.25 mg), common (0.5–1.5 mg), strong (2 mg), heavy (3 mg) (DrugWatch, 2018).

Alprazolam is described by recreational users as less stimulating but more euphoric than etizolam, more stimulating and equally euphoric as diazepam, but shorter-acting and more sedating than either of them (DrugWatch, 2018). Physical effects associated with the molecules use include “feelings of pleasure, relaxation and bodily comfort, disinhibition and reduction in anxiety” (DrugWatch, 2018).

Alprazolam may also be used to assist in dealing with dysphoria ‘bad trips’ resulting from hallucinogenic/psychedelic drug use (attendant distress or panic); ‘come-down’ effects of stimulant use (insomnia and agitation); enhancing the effects of other depressant drugs; enhancing the dissociative effects of drugs like ketamine or even generating a synergistic result with cannabis (DrugWatch, 2018; Newcombe, 2010).

As a benzodiazepine, alprazolam has the potential for physical and psychological dependence and misuse amongst those prescribed it. Its high-binding affinity, rapid onset of effect and potency (up to 20 times that of diazepam (Ashton, 2002; BNF, 2020)) makes abuse more attractive to those not prescribed it. Alprazolam appears more likely to be misused by those prescribed it with a substance use disorder history (Griffiths and Wolf, 1990). It is preferred over other benzodiazepines by those who use alcohol and opioids as it is considered more ‘rewarding’ and boosting their effects (Ciraulo et al., 1997; Iguchi et al., 1989; Longo and Johnson, 2000; Wolf et al., 1990).

## Recreational use in the UK

As alprazolam is not usually asked about in regular surveys of drug use, it is difficult to ascertain the extent of its recreational use. However, in the UK context, some limited information is available. Hockenhull et al. (2019) suggest that the estimated national prevalence rate for UK adults for non-medical use (NMU) of alprazolam was 0.08 (range 0.01–0.15)% with no statistically significant difference (*p* = 0.898) between males and females. NMU rates in the last 90 days were higher amongst those aged 16–24 (0.37%) compared to those aged 25–34 (0.14%) and those aged 35 or more (0.01%), *p* < 0.001. This suggests that about 26,000 individuals aged 16–24 had used alprazolam non-medically in the last 90-day period across the UK. The main reasons for NMU were: to treat a medical condition (54.9%), to get high (39.1%), to come down (26.8%), to prevent withdrawal (11.3%).

Greater use in younger age groups is also evident in the results of two other studies. The VICE website reported that an opportunistic survey about Xanax conducted using its VICE UK Snapchat channel (aimed mostly at 13- to 24-year-olds) received 85,000 UK responses, of whom 35% reported that they had friends who had taken Xanax (Ewens, 2018). *The Tab*’s 2020 Drugs Survey of students at 14 different UK universities revealed that Xanax was the ‘drug of choice’ for 3.7% (*n* = 576) of the 16,017 respondents (Mussen and Shead, 2020).

## Rationale for the study

Concern about the non-medical or recreational use of alprazolam was first expressed three decades ago (Juergens, 1991) and through to the early 2000s in the United States where it is widely prescribed (Lane et al., 2005). It was in the mid-2010s that other countries started to become worried about such use, for example, Australia (Schaffer et al., 2016); this trend continues today, for example, Singapore (Chan et al., 2020).

In Wiltshire, UK, in November 2008 there were media reports of alprazolam being mistaken for heroin. Similar warnings were issued by the Drug and Alcohol Action Teams in Brighton & Hove, Camden, and Reading in April 2009 (Ghodse et al., 2009).

In the UK context, since early 2018, concern has been increasingly expressed about the existence of illicit supplies of alprazolam destined for the country to meet what appears to be an increasing demand (O’Connor, 2018). Its illegal/recreational use is not only leading to dependence and treatment requests (Ward, 2019) but also to poisonings/overdoses (O’Connor, 2018). Many of the latter are proving to be fatal (Izundu, 2019; Phillips, 2019; Thompson, 2018). “The deaths are a very tiny tip of what is a very huge iceberg” according to the Northern Ireland Coroner Joe McCrisken (quoted in The Week, 2019).

Trying to understand the nature and extent of deaths in the United Kingdom related to alprazolam is important if successful attempts are to be made to reduce and prevent such occurrences.

## Materials and methods

### Statistics on UK alprazolam-related fatalities

**Figure 1.**
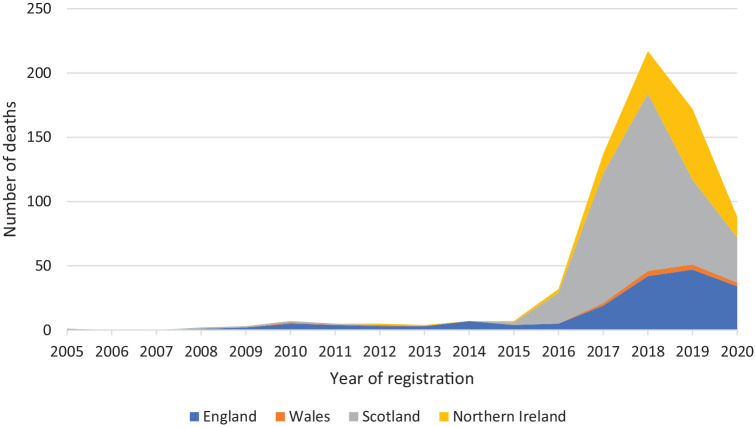
Alprazolam-related deaths registered in the United Kingdom, 2004–2020.

Whilst the three General Mortality Registers (GMRs) that cover the United Kingdom (Office for National Statistics (ONS) – England and Wales; National Records of Scotland (NRS); Northern Ireland Statistics & Research Agency (NISRA)) do publish annual information on drug deaths related to poisoning involving specific substances (Corkery, 2008), alprazolam is not one of those substances identified. However, some statistics have been released into the public domain following Freedom of Information (FOI) requests; additional data have been provided in response to requests by the lead author (JC). This information is given in Table 1 and Figure 1.

**Table 1.** Alprazolam-related deaths registered in the United Kingdom, by country and year, 2004–2020.

Year	England and Wales	England	Wales	Scotland	Northern Ireland	United Kingdom
2004	3	3	0	0	0	3
2005	1	1	0	0	0	1
2006	0	0	0	0	0	0
2007	0	0	0	0	0	0
2008	1	1	0	1	0	2
2009	2	2	0	1	0	3
2010	6	5	1	1	0	6
2011	4	4	0	1	0	5
2012	4	3	1	0	1	5
2013	3	3	0	0	1	4
2014*	8	7	0	0	0	8
2015	4	4	0	2	1	7
2016	5	5	0	24	3	32
2017	21	19	2	101	15	137
2018*	48	42	4	138	33	219
2019	51	47	4	66	55	172
2020*	39	34	3	35	16	90

Sources: NISRA – personal communications to JC 23 October 2020, 3 March 2021, 2 March 2022; ONS (2020c; 2021); NRS – EU-MADNESS project.

* Differences between the figures for England and Wales separately and England and Wales overall in 2014, 2018 and 2020 are because non-residents were excluded from the national figures. Numbers for Scotland do not necessarily agree with those published by NRS.

### Alprazolam-related events recorded on Scottish death certificates

Anonymised data on deceased individuals derived from death certificates and partial information from pathologists on substances found in post-mortem toxicology have been provided to JC by NRS as part of an agreement with the University of Hertfordshire for the European Commission-funded research project EU-MADNESS (see Acknowledgements). The data cover deaths registered during the period 1 January 2013 to 31 December 2020. Deaths considered for analysis were those where ‘alprazolam’ or ‘Xanax’ was mentioned in the ‘cause of death’ field in the medical death certificate and/or the list of substances considered by the pathologist in the index cases to have caused or contributed to the fatality (NRS, 2021a).

### Data analysis

Scottish data were examined in respect of the characteristics of decedents and deaths using descriptive statistics. Microsoft Excel 365 was used to produce these statistics. Seasonality differences were examined using the Ratio of Proportions in conjunction with a *X*^2^ (chi-square) test.

### Ethics

Ethical approval is not required in the UK for studies whose subjects are deceased, and solely involves retrospective reviews of death records.

## Results

### UK alprazolam-related deaths

To provide some context for the Scottish data, we present high-level UK statistics. According to relevant UK agencies, there were no deaths where alprazolam was mentioned in the ‘cause of death’ field on the medical certificate of cases officially registered during the following periods: England and Wales, 1993–2003; Scotland, 1996–2003; Northern Ireland, 1997–2003. The number of deaths where alprazolam was mentioned during the period from 2004 onwards are presented in Figure 1 and Table 1.

There was no more than an overall total of eight registered deaths in a single year across the United Kingdom between 2004 and 2015. However, since then the numbers published by the agencies or provided to JC have risen rapidly: 32 in 2016, 137 in 2017, 219 in 2018. There was an overall fall in 2019: England showed a slowing down in the rate of increase; Wales showed a levelling-off, whereas Scotland and Northern Ireland experienced rapid falls. All countries experienced a fall in 2020, albeit from a low level in Wales.

### Focus on deaths registered in Scotland

Data provided by NRS permit some analysis of characteristics of decedents and deaths registered during the period 2015–2020 (*n* = 366). Four-fifths (77.1%) of decedents were male; the mean age was 39.0 (SD 12.6) years (Table 2). The underlying cause of death in most cases was accidental poisoning, principally involving opiates/opioids (77.9%) and sedatives, hypnotics and similar substances (15.0%). Unsurprisingly, most deaths (93.2%) were accidental in nature, as opposed to being intentional (Table 2).

**Table 2.** Main characteristics of alprazolam-related deaths registered in Scotland, 2015–2020.

Characteristic	Number	Percentage
Gender		
All persons	366	100.00
Male	282	77.05
Female	84	22.95
Underlying cause of death (ICD-10)		
F13.2 (Mental and behavioural disorders due to use of sedatives or hypnotics – dependence syndrome)	2	0.55
F19.2 (Mental and behavioural disorders due to multiple drug use and use of other psychoactive substances – dependence syndrome)	1	0.27
I25.9 (Ischaemic heart disease)	1	0.27
V43.5 (Driver in vehicular collision)	1	0.27
W19 (Presumed fall)	1	0.27
W69 (Immersion in water)	1	0.27
X41 (Accidental poisoning by and exposure to antiepileptic, sedative-hypnotic, antiparkinsonism and psychotropic drugs, not classified elsewhere)	55	15.03
X42 (Accidental poisoning by and exposure to narcotics and psychodysleptics (hallucinogens), not classified elsewhere)	285	77.87
X44 (Accidental poisoning by and exposure to other and unspecified drugs, medicaments and biological substances)	1	0.27
X62 (Intentional self-poisoning by and exposure to narcotics and psychodysleptics (hallucinogens), not classified elsewhere)	1	0.27
Y11 (Poisoning by and exposure to antiepileptic, sedative-hypnotic, antiparkinsonism and psychotropic drugs, not classified elsewhere, undetermined intent)	3	0.82
Y12 (Poisoning by and exposure to narcotics and psychodysleptics (hallucinogens), not classified elsewhere, undetermined intent)	14	3.83
Manner of death		
Accidental (X41, X42, X44)	341	93.17
Suicide (X62)	1	0.27
Undetermined intent (V43.5, W19, W69, Y11, Y12)	20	5.46
Drug abuse (F13.2, F19.2)	3	0.82
Natural (I25.9)	1	0.27
Age (years)		
Mean: 38.99; range: 14–64; SD: 12.553		
Number of drugs implicated		
Mean: 4.77; range: 1–11; SD: 1.821		

The mean number of substances involved in deaths was 4.77 (SD 1.821). There were only two deaths where alprazolam was the only substance involved in the death (Table 3). Table 3 shows that the main drug groups implicated in death were: opiates/opioids (94.8%), other benzodiazepines (67.2%), gabapentinoids (42.9%), stimulants (30.1%), and antidepressants (15.0%). The other main drug groups mentioned were alcohol (7.4%) and antipsychotics (2.5%). The polysubstance nature of these deaths is reflected in the common combinations of drug classes implicated (Table 4). Two-thirds (64.2%) involved both other benzodiazepines and opiates/opioids; these drug groups are CNS depressants.

**Table 3.** Drugs most commonly implicated in alprazolam-related deaths registered in Scotland, 2015–2020.

Drug class	Selected drugs	Number	Percentage
Alprazolam mention	Sole	2	0.55
	Any	366	100.00
Other benzodiazepines	Of which	246	67.21
	Diazepam	176	48.09
	Etizolam	82	22.40
	Delorazepam	13	3.55
	Phenazepam	8	2.19
	Lorazepam	7	1.91
	Diclazepam	6	1.64
	Flualprazolam	6	1.64
	Temazepam	3	0.82
	Nitrazepam	2	0.55
	Adinazolam	1	0.27
	Clonazepam	1	0.27
	Cloxazolam	1	0.27
Alcohol	Any	27	7.38
Antidepressants	Of which	55	15.03
	Mirtazapine	33	9.02
	Amitriptyline	15	4.10
	Sertraline	4	1.09
	Fluoxetine	3	0.82
	Venlafaxine	2	0.55
	Nortriptyline	1	0.27
Antipsychotics	Of which	9	2.46
	Olanzapine	6	1.64
	Quetiapine	2	0.55
	Risperidone	1	0.27
Gabapentinoids	Of which	157	42.90
	Pregabalin	108	29.51
	Gabapentin	67	18.31
Opiates/opioids	Of which	347	94.81
	Methadone	234	63.93
	Heroin/morphine	187	51.09
	Heroin	127	34.70
	Morphine	93	25.41
	Dihydrocodeine	40	10.93
	Buprenorphine	30	8.20
	Tramadol	16	4.37
	Codeine	14	3.83
	Oxycodone	9	2.46
	Fentanyl	8	2.19
	Hydrocodone	4	1.09
Stimulants	Of which	110	30.05
	Cocaine	99	29.46
	Amfetamine/amphetamine	9	2.46
	MDMA/MDA	6	1.64
	Modafinil	1	0.27

MDA: 3,4-methylenedioxyamphetamine; MDMA: 3,4-methylenedioxymethamphetamine.

**Table 4.** Main combinations of drug classes implicated in alprazolam-related deaths registered in Scotland, 2015–2020.

Combination (selected)	Number	Percentage
Other benzodiazepines + opiates/opioids	235	64.21
Other benzodiazepines + stimulants	82	22.40
Other benzodiazepines + opiates/opioids + stimulants	78	21.31
Other benzodiazepines + opiates/opioids + antidepressants	35	9.56
Opiates/opioids + alcohol	23	6.28

**Figure 2.**
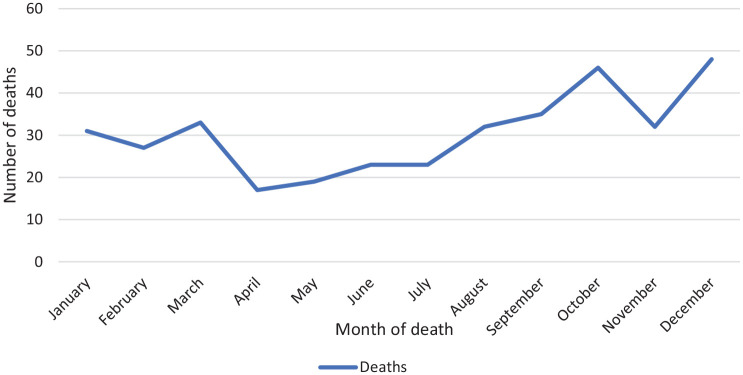
Alprazolam-related deaths in Scotland, by month of occurrence, aggregated data, 2015–2020.

**Figure 3.**
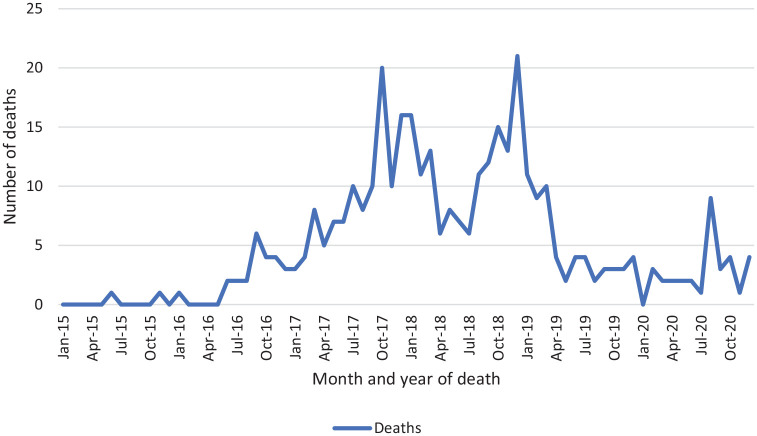
Alprazolam-related deaths in Scotland, by month of occurrence, 2015–2020.

The number of deaths is lower during the ‘spring’ and ‘summer’ months March to August (*n* = 147), compared with the ‘autumn’ and ‘winter’ months September to February (*n* = 219) – see Figure 2. This difference is statistically significant (Ratio of Proportions, *p* < 0.0001, *Χ*^2^ critical value = 28.289, CI = 12.4605, 26.5948). This seasonality can also be seen in the disaggregated data presented in Figure 3.

## Discussion

This section looks at the main issues emerging from the above analyses. Comparisons are made, where possible, with other relevant research findings.

### Trends in numbers of alprazolam-related deaths over time

There is no comprehensive international overview of alprazolam deaths over time. To see how Scotland and the United Kingdom fit more generally into the wider picture, we have attempted to provide an overview, based on published papers available to us.

The first death involving alprazolam reported in the international literature appears to have occurred in 1983–1984 (Edwards et al., 1991). This mention was followed by several papers in following years, usually dealing with a sole case report or small case-series reports (e.g. Feola et al., 2021; Haque et al., 1990; Jenkins et al., 1997; McIntyre et al., 2017; Michaud et al., 1999; Rogers et al., 1997; Tungtananuwat and Lawanprasert, 2010). These were typically related to the use of alprazolam in conjunction with other substances (typically opioids), although one case report concerns a death associated with withdrawal from alprazolam (Haque et al., 1990).

The first large-scale studies identifying alprazolam-related deaths started in the early 2000s, but were chiefly concerned with documenting deaths from all drugs (Austin et al., 2017; Goldberger et al., 2011; Haukka et al., 2017; Hedegaard et al., 2018; Ossiander, 2014; Simonsen et al., 2021), other drug classes (Bishop-Freeman et al., 2021; Chan et al., 2006; Jönsson et al., 2007; Mariottini et al., 2021), psychotropic drugs (Pfeifer et al., 2019) or benzodiazepines (Kriikku et al., 2020), with such deaths being incidentally uncovered. On the other hand, some studies had alprazolam deaths as their primary focus (Darke et al., 2014; Jones and Holmgren, 2013; Rintoul et al., 2013; Shah et al., 2012; Wolf et al., 2005). In addition, some studies have looked at the toxicity of alprazolam compared to other drugs (Ojanperä et al., 2016), benzodiazepines (Isbister et al., 2004; Skov et al., 2016), other antidepressants (Buckley and McManus, 2004; Jönsson et al., 2014) or ‘z’ drugs (Reith et al., 2003).

Overall, deaths from the consumption of alprazolam alone are thought to be rare (Wolf et al., 2005). Consumption of the medication with other CNS depressants, such as alcohol, other benzodiazepines, gabapentinoids and opiates/opioids, increases the likelihood of overdose through respiratory depression.

Edwards et al. (1991) recorded one successful intentional overdose amongst a cohort of 10,895 patients on alprazolam followed up for an average of 3.7 months during 1983/1984 in England. This seems to be the first reported death involving the medication in the UK. Buckley and McManus (2004) note three deaths in Great Britain (England, Wales and Scotland) involving alprazolam alone or alcohol during the period 1983–1999.

Between 2004 and 2015, no more than eight deaths were registered annually in the UK. However, numbers rose to 32 in 2016, 137 in 2017, peaking at 219 in 2018. Whilst data are lacking for Scotland in terms of the availability of illicit supplies of alprazolam, which might partially explain the increase in deaths, data from the rest of the UK do suggest large shipments being intercepted (Border Force responses on 25 January 2021 to FOI request 61791 and 24 March 2021 to FOI request 62855 by JC, and personal communication to JC from the Police Service of Northern Ireland on 27 November 2020). However, it should also be noted that the increase in alprazolam-related fatalities should be seen against the dramatic increase in numbers of deaths in Scotland involving benzodiazepines more generally (McAuley et al., 2022).

Substantial falls occurred in both 2019 and 2020 (to 172 and 90, respectively). The number of deaths registered in England continued to rise in 2019, albeit at a slower rate, but fell back in 2020. Wales experienced a levelling-off in the last couple of years. The number of deaths in Northern Ireland reached a new peak in 2019 before falling back to 2017 levels in 2020. By contrast, Scotland has experienced rapid falls since 2018, although still above the level recorded in 2016.

The falls in the numbers of alprazolam-related deaths in England (ONS, 2021), Scotland (NRS, 2021a) and Northern Ireland (NISRA, 2022b) may well have been offset by increasing number of cases registered in 2019 and 2020 involving flualprazolam and other designer benzodiazepines, especially etizolam (e.g. see McAuley et al., 2022). There have been some local initiatives in Scotland alerting prescribers, clinicians and users of alprazolam about its potential lethality, especially in conjunction with other CNS depressants. Examples include NHS Grampian (2017), Borders Alcohol & Drugs Partnership (2018), Horne (2018), with similar alerts issued in England (Mahase, 2020; Sky News, 2020). However, the timing of such releases is difficult to map onto the patterns in fatalities. These initiatives may have had an impact on drug users. That said, similar warnings about designer benzodiazepines do not appear to have had the desired impact. COVID-19 could have played a part in the decreasing role of alprazolam in drug-related deaths, for example, by lockdown measures limiting access to illicit supplies (Bergeron et al., 2020; Whitfield et al., 2021) or to prescribed benzodiazepines (Carr et al., 2021), or reduced access to treatment (Carr et al., 2021; Di Gessa et al., 2022) leading to self-medication with substitute drugs (Zaami et al., 2020). Further investigation is warranted into the fall in alprazolam-related deaths.

Whilst the overall mean proportion of all drug-poisoning deaths accounted for by alprazolam during the period 2015–2020 was 0.664% in England and 0.833% in Wales, the corresponding figures for Scotland and Northern Ireland were 5.203% and 12.239%, respectively (see Table 5). Indeed, at the peak of alprazolam-related deaths registered in 2018, 1 in 10 and 1 in 6 of all drug-poisoning deaths in Scotland and Northern Ireland, respectively, were accounted for by this drug. Moreover, in 2019 this proportion increased to more than one in four in respect of Northern Ireland. These national differences reflect already recognised patterns in terms of higher rates of benzodiazepines being implicated in drug-poisoning deaths in Scotland and Northern Ireland (Corkery et al., 2020), and the much higher drug-poisoning death rate per 100,000 population in Scotland compared to other parts of the United Kingdom and Europe (PHE, 2020). However, the proportion of deaths in Northern Ireland accounted for by alprazolam is unexpectedly higher than one might expect in comparison with Scotland.

**Table 5.** Proportion of all drug-poisoning deaths where alprazolam was implicated, by country within the United Kingdom, 2015–2020.

Year(s)	Country			
Category of death	England	Wales	Scotland	Northern Ireland
Alprazolam-related	Number			
2015	4	0	2	1
2016	5	0	24	3
2017	19	2	101	15
2018	42	4	138	33
2019	47	4	66	55
2020	34	3	35	16
2015–2020	151	13	366	123
All drug-poisoning deaths	Number			
2015	3416	238	813	144
2016	3450	271	997	127
2017	3482	260	1045	136
2018	3983	327	1313	189
2019	4115	240	1406	191
2020	4312	224	1461	218
2015–2020	22,758	1560	7035	1005
	Percentage			
2015	0.117	0.000	2.460	0.694
2016	0.144	0.000	2.407	2.362
2017	0.546	0.769	9.665	11.029
2018	1.054	1.223	10.510	17.460
2019	1.142	1.667	4.694	28.796
2020	0.788	1.339	2.396	7.339
2015–2020	0.664	0.833	5.203	12.239

Drug-poisoning deaths are defined as those where the underlying cause of death has been classified using the following International Classification of Diseases, Tenth Revision (WHO, 1992): F11–F16, F18–F19, X40–X44, X60–X64, X85, Y10–Y14.

Alprazolam-related deaths in the Republic of Ireland increased from less than 5 in each of the years 2004–2009 to 12 in 2010, before climbing to 47 in 2014 and remaining at that level until an increase of 34% to 63 in 2017 (Lynn and Lyons, 2019a, 2019b). The increase in deaths involving alprazolam in Ireland appears to have occurred several years earlier than in other parts of the British Isles, including Northern Ireland. However, Ireland experienced a major increase between 2016 and 2017 as did the constituent parts of the UK. These patterns are later than the increases experienced elsewhere, for example, Australia and the United States.

In the United States, Florida experienced a 234% increase in the rate of alprazolam deaths from 1.3 in 2003 to 4.4 in 2009 per 100,000 population (Goldberger et al., 2011). Palm Beach County saw an increase in the number of cases where alprazolam was found in post-mortem toxicological investigations from 45 in 2001 to 83 in 2002, with 50 in the first 8 months of 2003 (Wolf et al., 2005). West Virginia experienced a 346% increase in the number of deaths in which alprazolam was found in post-mortem toxicology from 26 in 2005, to 62 in 2006 and 116 in 2007 (Shah et al., 2012). At a national level, alprazolam was outside the top 10 drugs involved in drug overdose deaths in 2010 (Warner et al., 2016). However, the following year it made its appearance at number 5 and remained around that position up to and including 2016, appearing in between 8.5% and 9.8% (numbers varying between 3724 and 6209) of all such deaths (Hedegaard et al., 2018).

In Australia, New South Wales registered an increase from two or three deaths p.a. in 1997–1999 in which alprazolam was involved to 12 in 2005, with a sharp increase starting in 2009 (57 deaths) reaching 86 in 2012 (Darke et al., 2014). Whilst Darke et al. (2014) found no statistically significant variance by month (*p* = 0.17), in Scotland we found a seasonal effect (*p* < 0.0001).

### Socio-demographics

Although there are no age and gender breakdowns for UK alprazolam-related deaths, Scottish data indicate that, over the period 2015–2020, males accounted for 77.05% of such cases and the mean age was 38.99 (range: 14–64; SD: 12.553) years.

It appears that the Scottish deaths are more skewed towards males when compared to other studies: 66.5% (Darke et al., 2014), 69.1% (Shah et al., 2012), but is close to the 74.9% reported by Wolf et al. (2005) and the 82.1% reported by Haukka et al. (2017). However, the mean age at death falls between those established by Shah et al. (2012) (mean: 37.7, SD: 10.8) and Darke et al. (2014) (mean: 41.3, SD: 13.7, range: 17–90), but is somewhat higher than the value of 35 years found by Haukka et al. (2017) and the mean of 36.3 (range: 16–84) years found by Wolf et al. (2005).

### Cause(s) and manner of death

Wolf et al. (2005) reported that 48.9% of deaths (total *n* = 178) with a positive post-mortem alprazolam finding were due to combined drug toxicity. The drug was considered an incidental finding in 44 traumatic deaths, 12 natural cause deaths and 33 cases attributed to other drug(s). Drug toxicity accounted for two-thirds (67.4%) of deaths in the study by Darke et al. (2014); 57.0% accidental, 10.4% deliberate. Other means of suicide accounted for 12.6% (hanging 24/52 cases), disease 10.0% (41 cases, of which cardiovascular 25, pulmonary 12), accident 5.1% (15/21 motor vehicle accidents), homicide 2.4% and unascertained 2.4%. Drug toxicity, often in polysubstance scenarios, features highly in both of these studies, echoing the Scottish patterns. This trend has been observed across the world over the past couple of decades and is now a permanent feature of psychoactive drug consumption and deaths.

Shah et al. (2012) found that 90.2% of alprazolam-related deaths were accidental in nature, whilst 2.0% were suicidal; intentionality could not be established in 7.8% of cases. A slightly different pattern was observed in 87 deaths considered by Wolf et al. (2005) to have been due to combined drug toxicity: accidental 86.2%, suicidal 10.3% and undetermined intent 3.4%. These sets of results are broadly in line with the Scottish deaths: 93.2% deemed accidental, 0.3% suicidal and 5.5% of undetermined intent. These findings, that is, the majority of deaths being accidental in nature, underline the fact that many of these deaths were preventable.

### Numbers of drugs and substance types involved

The mean number of substances involved in Scottish alprazolam-related deaths was 4.77 (SD 1.821). Shah et al. (2012) reported the mean number of drugs implicated in alprazolam-related deaths was 2.9 (SD 1.0), higher than the 2.3 (SD 1.3) in non-alprazolam cases. Warner et al. (2016) reported an average of 2.3 additional drugs in alprazolam-related deaths.

There were only two (0.55%) Scottish deaths where alprazolam was the only substance involved in the death (Table 3). No deaths in Northern Ireland have involved alprazolam on its own, although in one instance in 2019 a death also implicated alcohol without any other substances (personal communication to JC from NISRA, 2 March 2022). Shah et al. (2012) recorded that alprazolam alone was implicated in only 5/193 (2.6%) alprazolam-related deaths. Wolf et al. (2005) reported that only two deaths were attributed to alprazolam alone in 178 post-mortem findings. Warner et al. (2016) report that 95.5% of US alprazolam-related deaths involved both it and other drugs during 2010–2014; this increased to 96.2% in 2016 (Hedegaard et al., 2018). These findings confirm previous claims that alprazolam alone causes death very rarely.

The main drug groups implicated in death in this present study were opiates/opioids (94.8%), other benzodiazepines (67.2%), gabapentinoids (42.9%), stimulants (30.1%) and antidepressants (15.8%). The other main drug groups mentioned were alcohol (7.4%) and antipsychotics (2.5%). Where positive post-mortem alprazolam findings were observed by Wolf et al. (2005) and the deaths were attributed to combined drug toxicity, the most common drugs implicated were cocaine (69.0%), methadone (56.3%), morphine (18.4%) and heroin (13.8%).

The most common groups of drugs co-ingested with alprazolam in the study by Shah et al. (2012) were opiates/opioids, mainly hydrocodone, methadone and oxycodone (88.7%); other benzodiazepines, mainly diazepam (37.7%); alcohol (13.2%) and antidepressants (11.3%). In 2014, the most common concomitant drugs in US alprazolam-related deaths were oxycodone (29.6%) and heroin (19.8%) (Warner et al., 2016); in 2016, the drugs involved were fentanyl (28.3%), heroin (26.9%) and oxycodone (25.3%) (Hedegaard et al., 2018). These changes probably reflect the patterns of drug use in the United States when the studies were conducted.

The polysubstance nature of the Scottish deaths is reflected in the common combinations of drug classes implicated (Table 4). Two-thirds (64.2%) involved both other benzodiazepines and opiates/opioids; these drug groups are CNS depressants.

Most of the alprazolam-related deaths in Ireland during 2016 and 2017 involved other drugs: 2016 – methadone, 22; diazepam, 22; heroin, 14; alcohol, 12; 2017 – methadone, 42; diazepam, 36; heroin, 29; alcohol, 9 (Lynn and Lyons, 2019a, 2019b). These findings echo those outlined above for Scotland.

Research focusing on deaths attributed to other classes of drugs indicates that alprazolam is often associated. Chan et al. (2006) in their study of methadone-positive deaths found that the drug was present in 47.7% of accidental overdose fatalities and 39.3% of deaths from all other causes. Mariottini et al. (2021) found that alprazolam was present in 31.9% of buprenorphine user deaths. Rintoul et al. (2013) found that during the period 1996–2010, in the Australian state of Victoria, the proportion of heroin-related deaths in which alprazolam was detected rose from 1.4% to 28.1%. Ossiander (2014) found that in deaths involving Washington state residents in 2003–2010 where methadone was present, 8.6% also had alprazolam; the proportion of deaths where alprazolam was present was 10.9% for oxycodone, and 2.2% for heroin.

### Toxicity of alprazolam relative to other drugs

Several studies have compared the toxicity of alprazolam to that of other drugs, including other benzodiazepines. Using a Fatal Toxicity Index (FTI), Reith et al. (2003) concluded that alprazolam (38.1) had a higher death rate than chlormethiazole (24.7) per 100,000 prescriptions, but with a similar rate (16.0) to clonazepam (16.1) but far lower than chlormethiazole (55.4) per million defined daily doses for statistical purposes (S-DDD). Using a similar approach, Buckley and McManus (2004) found that for drugs used primarily as anxiolytics, alprazolam had a lower death rate per million prescriptions of 7.5 compared to bromazepam (8.9) and meprobamate (19.0), but a higher rate than other benzodiazepines.

Ojanperä et al. (2016) calculated the mean FTI, based on the number of deaths per million defined daily dosdes (DDD)s, for 70 medicinal drugs in Finland in the years 2005, 2009 and 2013. Alprazolam was ranked 27th with a rate of 1.02; this compares to a rate of 42.65 for methadone, 0.52 for temazepam and 0.16 for nitrazepam. Based on an examination of post-marketing reports of medications in the United States between 1968 and 2017, Marwitz et al. (2020) found that alprazolam was 12th in the list; 72.4% of reports were from poison control centres.

Using the ratio of sole mention to any mention of an index substance in the ‘cause of death’ field on medical death certificates (King and Corkery, 2018), it is possible to compare the toxicity of alprazolam to other benzodiazepines in Scotland for the period 2013–2020. Table 6 shows that alprazolam has a ratio of 0.005 compared to 0.007 for etizolam, 0.012 for delorazepam, 0.016 for flualprazolam, 0.019 for flubromazolam and 0.057 for temazepam. This means it has a lower inherent toxicity on its own than the other specified molecules. The ratios for benzodiazepine analogues and all benzodiazepines were 0.007 and 0.009, respectively. It should be noted that some of the frequencies for some benzodiazepines are relatively low. The ratio for benzodiazepine analogues (0.007) is somewhat lower than that found (0.045) by King and Corkery (2018) for England and Wales during the period 2009–2016.

**Table 6.** Ratio of sole/any mentions where specific benzodiazepines were implicated in the cause of death registered in Scotland, 2013–2020.

Drug name	Total number of deaths		Mean number of deaths per year		Ratio of sole/any
	Sole mention	Any mention	Sole mention	Any mention	
Alprazolam	2	366	0.250	45.750	0.005
Adinazolam	0	1	0.000	0.125	0.000
Benzodiazepine(s)	6	278	0.750	34.750	0.022
Bromazepam	0	4	0.000	0.500	0.000
Chlordiazepoxide	0	9	0.000	1.125	0.000
Clobazam	0	1	0.000	0.125	0.000
Clonazepam	0	7	0.000	0.875	0.000
Cloxazolam	0	15	0.000	1.875	0.000
Delorazepam	1	83	0.125	10.375	0.012
Diazepam	4	1339	0.500	167.375	0.003
Diclazepam	0	178	0.000	22.250	0.000
Etizolam	21	2879	2.625	359.875	0.007
Flualprazolam	1	64	0.125	8.000	0.016
Flubromazepam	0	18	0.000	6.000	0.000
Flubromazolam	1	53	0.125	6.625	0.019
Lorazepam	0	26	0.000	3.250	0.000
Lormetazepam	0	2	0.000	0.250	0.000
Midazolam	0	1	0.000	0.125	0.000
Nitrazepam	0	9	0.000	1.125	0.000
Nordiazepam	0	1	0.000	0.125	0.000
Oxazepam	0	17	0.000	2.125	0.000
Phenazepam	1	183	0.125	22.875	0.005
Pyrazolam	0	4	0.000	0.500	0.000
Temazepam	3	53	0.375	6.625	0.057
Benzodiazepine analogues	23	3111	2.875	388.875	0.007
All benzodiazepines	40	4256	5.000	532.000	0.009

Eight years of exposure; benzodiazepine analogues = diclazepam, eitzolam, flubromazepam, flubromazolam, phenazepam, pyrazolam (King & Corkery, 2018).

### Fatal toxicity levels for alprazolam

Although this study did not have access to post-mortem toxicology levels in the Scottish alprazolam-related fatalities described here, some information on Scottish alprazolam-positive cases is available (O’Connor, 2020). This is presented here so readers have easy access to it. The mean concentration of alprazolam in 232 cases screened in 2016–2018 by the Forensic Toxicology Service, University of Glasgow, was 0.179 mg/L. The cases had a mean age of 39 years; 77% were males.

Further limited information has been published on fatal levels of alprazolam (Table 7).

**Table 7.** Published post-mortem alprazolam toxicology levels.

Study	Alprazolam	Other substances detected	Comments
Wolf et al. (2005)	Blood levels: trauma related (*n* = 44) – mean 117.0 (range 2–998) ng/mL; natural causes (*n* = 12) – mean 41.0 (range 2–82.0) ng/mL; other drugs (*n* = 33) – mean 43.0 (range 4–130) ng/mL	Not reported	Due to a wide range of post-mortem alprazolam blood levels, it was difficult to assess the role of the drug in deaths
Darke et al. (2014)	Overall median blood levels 0.08 (range 0.005–2.10) mg/L; intentional overdoses 0.18 mg/L; accident 0.09 mg/L; accidental drug overdose 0.08 mg/L; accident deaths where it was a significant factor 0.20 mg/L	Not reported	
Jönsson et al. (2014)	Median femoral blood concentrations: 0.30 μg/g where the sole drug causing death; 0.16 for deaths involving two or more drugs; 0.05 in a control group and driving cases; 0.04 in therapeutic drug monitoring cases	Not reported	μg/g can be converted to μg/mL using a multiplier of 1.06, the average density of blood
Skov et al. (2016)	Femoral blood levels: mean of 0.11 (range 0.076–0.15 mg/kg) for two deaths where a contributing cause of death; mean of 0.025 (median 0.024, range 0.002–0.06 mg/kg) for 12 cases where unrelated to cause of death	Not reported	
Ketola and Ojanperä (2019)	Femoral blood concentrations in all cause deaths (*n* = 2234): mean 0.006 mg/L; median 0.033 mg/L	Not reported	
Jones and Holmgren (2013)	Median blood levels were similar in impaired drivers (0.05 mg/L) to both intoxication deaths (0.06 mg/L) and other causes of death (0.05 mg/L); mean levels were (0.08, 0.10 and 0.08 mg/L, respectively)	Not reported	
Michaud et al. (1999)	Blood concentration 0.21 mg/L	Alcohol 1.29 g/kg; tramadol 38.3 mg/L	
Tungtananuwat and Lawanprasert (2010)	Blood level of 0.2 μg/mL	Tramadol 0.27 μg/mL; nortriptyline 1.78 μg/mL; methadone 0.3 μg/mL; methamphetamine 0.4 μg/mL; caffeine 0.39 μg/mL	
McIntyre et al. (2017)	Peripheral blood concentration 0.12 mg/L	U-47700 340 ng/mL; nordiazepam <0.05 mg/L; doxylamine 0.30 mg/L; diphenhydramine 0.14 mg/L; ibuprofen 2.4 mg/L; salicylic acid <20 mg/L; 11-nor-9-carboxy-delta-9-tetrahydrocannabinol 2.4 ng/mL	Death due to acute abuse of the synthetic opioid U-47700 and alprazolam
Feola et al. (2021)	Alprazolam and metabolite α-hydroxyalprazolam (respectively) concentrations: femoral blood 0.45 and 0.03 mg/L; urine 2.12 and 0.42 mg/L; bile 1.33 and 0.56 mg/L; liver 3.81 and 0.28 mg/L; alprazolam alone in vitreous humour (0.19 mg/L) and in stomach contents (0.34 mg/L)		A fatal poisoning by alprazolam in a 60 years old male was considered an alprazolam drug-related death based on the decedent’s pre-existing bronchopneumonia and slight hypertrophy of the left ventricle; levels of alprazolam several times greater than the therapeutic range contributed to death due to its respiratory and CNS-depressant effects.

Blood levels vary according to the way the researchers have categorised the role of alprazolam in the reported cases and the units used to present data; this makes comparisons very difficult. However, what we can state is that in the few cases where alprazolam was the sole drug causing death, a median value of 0.30 μg/g or 31.8 mg/L (and 0.45 mg/L in one case) was reported. This contrasts to deaths in which alprazolam was implicated with other substances where levels were mean 43.0 (range 4–130) ng/mL; median 0.16 μg/g or 16.96 mg/L.

Wolf et al. (2005) give ante-mortem blood levels of 32.8 and 13.0 ng/mL for two alprazolam toxicity cases. By comparison, the ante-mortem serum levels of two unsuccessful suicide attempts involving alprazolam were reported as 350 and 341 ng/mL (McCormick et al., 1985). Steady-state plasma levels following alprazolam therapy have been reported as being in the range of 20–55 ng/mL (Abernethy et al., 1983; McCormick et al., 1984).

### Alprazolam availability and death

It could be reasonably argued that the greater the supply and/or availability of alprazolam, the likelier the occurrence of fatalities associated with its use. Unfortunately, there is very little research looking at this relationship – in fact, just one study. Focussing on heroin-related deaths between 1996 and 2010 in the Australian state of Victoria, Rintoul et al. (2013) noted not only an increased proportion of deaths where alprazolam was detected (1.4%–28.1%) but also in the number of prescriptions (95,000–240,000), number of prescriptions per 100,000 population (2087–4327) and DDD per 1000 population per day (2.06–6.41).

The increases in alprazolam-related deaths in the UK up to 2018 cannot be explained by looking at availability of alprazolam in terms of amounts prescribed. No alprazolam is prescribed in primary care, and only very small amounts are prescribed via secondary or tertiary care. Unfortunately, no information is available on private prescribing of the drug in the United Kingdom.

The international literature warns against prescribing alprazolam to particular ‘at-risk’ groups, including those with an increased risk of deliberate self-poisoning (Austin et al., 2017; Isbister et al., 2004); those with a clinical history of substance abuse (Darke et al., 2014); polysubstance drug users (Darke et al., 2014), especially those taking opioids (Bannon et al., 2021; Ojanperä et al., 2016).

We have to look for other explanations for this increase in UK deaths. Very large amounts of alprazolam/Xanax tablets are being intercepted by UK law enforcement agencies. This strongly implies that illegal vendors/suppliers are meeting a demand that cannot be met by diversion from the very small licit supply within the UK. Alprazolam is easily available from online pharmacies (Zhao et al., 2020), and on the dark web (Lee, 2018). We are unaware of any diversion from the legal supply chain in the United Kingdom, although police have advised vets about possible thefts (RCVS, 2009). However, there are examples in other countries (Keith, 2019; Ove, 2018), as well as instances of thefts (McKnight, 2020; WTO, 2012), robbery (Heylin, 2021) and forgery (Department of Justice, 2020).

NMU of alprazolam appears to be on the increase globally. Factors playing a role in this development include: a known history of drug abuse (Haukka et al., 2017); NMU of prescription drugs (Haukka et al., 2017). These changes need to be seen against increasing trends in the NMU of benzodiazepines globally (UNODC, 2017) and ‘designer benzo’ use in the United Kingdom (ACMD, 2020). This has been well recognised in Scotland (Johnson et al., 2016; O’Connor, 2020).

Unparalleled levels of deaths involving such benzodiazepines are occurring in UK, especially in Scotland (e.g. etizolam) and, more recently, in Northern Ireland, because of NMU.

Recreational drug users are attracted to alprazolam because of its high lipophilicity which leads to faster bioavailability, especially in the context of crossing the bloodbrain barrier and thus a quicker ‘hit’. Compared to other benzodiazepines, alprazolam has a greater potency (Ashton, 2002; BNF, 2020).

### Strengths and limitations of the study

This is the first UK study to look at the nature and extent of alprazolam-related deaths, and it also provides the first and most up-to-date overview of research into alprazolam-related deaths. Furthermore, it highlights the need for healthcare professionals to be aware of the scale of illicit use of alprazolam.

There are several limitations to the Scottish data here which precludes further in-depth analyses. The NRS does not receive detailed toxicological information, only an indication from the relevant pathologist as to what were the substances considered to have been implicated in and/or contributing to death, as well as an indication of other substances found at post-mortem. No levels in body tissues are provided. The equivalent agencies that cover England and Wales (ONS) and Northern Ireland (NISRA) do not receive any toxicological information. The number of deaths presented in Figure 1 and Table 1 only relate to deaths due to drug poisonings (Corkery, 2008). Whilst National Programme on Substance Abuse Deaths (NPSAD) data would be complementary, this is outside the remit of the current paper as NPSAD has not received Scottish data since 2011. A future study could be carried out to compare/contrast Scottish trends with English/Welsh/Northern Irish trends using NPSAD data.

The death certificate literal or textual ‘cause of death’ information has been used by other researchers investigating and/or reporting alprazolam-related deaths (Hedegaard et al., 2018; Ossiander, 2014). This approach has been widely used to look at drug-poisoning deaths both internationally (Trinidad et al., 2016) and in the UK context by JC (see e.g. Corkery, 1997; Corkery et al., 2018; Schifano et al., 2008).

The data published by the UK GMRs are based on the year that a death is registered rather than the year of occurrence; this provides timely and consistent time series. However, this means that, for true epidemiological purposes, such statistics are not as useful for looking at year-on-year changes or to evaluate sudden changes (ONS, 2020b). That said, typically, the overall trends are similar; a comparison (unpublished data) between these two types of event for the Scottish data presented here shows only a slight shift forward in time.

Whilst there is, usually, only a relatively short period between a death occurring and it being registered, the actual determination of the cause of death and whether drugs or other substances were involved can take months (ONS, 2020a). In England and Wales, and to a lesser extent in Northern Ireland, coronial inquests take an average of 27 weeks to be completed once a death is notified (Ministry of Justice, 2021; NISRA, 2022a). Similar investigations are undertaken by the Crown Office and Procurator Fiscal Service in Scotland (NRS, 2021b). This means that the (ICD-10) coding of the death for statistical purposes takes place a considerable time after the death was first registered.

Prescribing and substance use histories are also unavailable to these agencies. Thus, it is impossible to know, without linkage to prescribing and/or drug-treatment records, whether a decedent had consumed alprazolam prescribed to them in the period leading up to death. Such information could only be obtained through examining Coronial or Procurator Fiscal records, record linkage with prescribing records, or psychological autopsies.

## Conclusions

This paper reports the first study looking at alprazolam-related deaths in the UK. It provides hard facts about the number of alprazolam deaths, that is, as an indicator of the extent of the problem in terms of its worst outcome, and the ways in which alprazolam contributed to deaths. The main themes emerging are outlined below.

Misuse and recreational use of alprazolam are still prevalent. More individuals are becoming dependent on the drug and are seeking treatment. This phenomenon is reflected in the number of deaths where alprazolam is implicated with other substances. On the other hand, alprazolam is only rarely implicated as the sole substance in drug-poisoning death; it is a relatively safe medication when used as indicated. However, it would be helpful, in the UK context, if there was a mechanism in place to collate data on alprazolam scripts from second and tertiary care, as well as of private prescriptions.

There are several key messages emerging from this which, mainly, underline what some of the existing literature has suggested. When deciding whether to give a patient a script for alprazolam, prescribers should consider any history of substance abuse, especially of prescribed medications; polysubstance use; other medications prescribed, especially CNS depressants; suicidal ideation and suicide attempts. Pharmacists dispensing the drug can monitor amounts supplied so that excessive quantities for those at risk of suicide are avoided and supervise consumption in in-patient settings (Carpenter et al., 2021). There is a clear need to improve the training of specialists and to highlight possible flaws in current teaching pathways, identify the main benefits and disadvantages regarding current use of benzodiazepines in clinical practice (Dell’Osso et al., 2015). More direct evidence of the extent of the deaths needs to be brought to the attention of prescribers, treatment providers, emergency department doctors and clinical toxicologists. This could be achieved through brief communications via professional journals or perhaps specific alerts/announcements/briefings from agencies such as Public Health Scotland, Public Health England, etc.

Those prescribed alprazolam or individuals considering acquiring it illicitly for recreational purpose should be aware of the dangers of using the drug with other substances, especially opiates/opioids and other benzodiazepines. Recreational users should be aware that tablets sold as Xanax may contain a variable amount of alprazolam (if any) as well as potentially fatal cutting agents (Blakey et al., 2021; Tobias et al., 2021; Wedinos, 2018, 2021). Public health alerts by NHS agencies and the police were aimed at both clinicians and drug users. More targeted education of drug users via social media, treatment providers could be initiated. The benzodiazepine street market has been transformed, not just in Scotland but elsewhere, in less than 10 years from one based on diversion of prescription drugs to ‘designer benzos’. This has increased the ‘risk environment’ (Rhodes, 2009) for benzodiazepine users, making it more difficult to minimise harms (Robertson et al., 2021).

Future research in the UK context should look at alprazolam-related deaths in relation to prescribing and treatment history, previous substance abuse and mental health history. This would entail using appropriate information from coronial investigations as employed by the National Programme on Substance Abuse Deaths or linked databases as in the case of the Scottish Drug-Related Deaths Database. Toxicological data would also be available from such sources to investigate what levels of alprazolam were considered fatal, on their own and in combination with other substances, and how that compares with other research findings outlined above.

It is also important to understand the factors that may have been at work in causing the fall in alprazolam-related deaths recorded in 2019 and 2020, and in the provisional data for 2021 available to the lead author. This would help public health officials, clinicians and others get an insight into what factors can be addressed for outbreaks that may happen with other drugs in the future.
